# Ribocentre: a database of ribozymes

**DOI:** 10.1093/nar/gkac840

**Published:** 2022-09-30

**Authors:** Jie Deng, Yaohuang Shi, Xuemei Peng, Yuanlin He, Xiaoxue Chen, Mengxiao Li, Xiaowei Lin, Wenjian Liao, Yuanyin Huang, Taijiao Jiang, David M J Lilley, Zhichao Miao, Lin Huang

**Affiliations:** Guangdong Provincial Key Laboratory of Malignant Tumor Epigenetics and Gene Regulation, Guangdong-Hong Kong Joint Laboratory for RNA Medicine, Sun Yat-Sen Memorial Hospital, Sun Yat-Sen University, Guangzhou 510120, China; School of Life Science and Technology, ShanghaiTech University, Shanghai, 201210, China; Guangzhou Laboratory, Guangzhou International Bio Island, Guangzhou 510005, China; Guangdong Provincial Key Laboratory of Malignant Tumor Epigenetics and Gene Regulation, Guangdong-Hong Kong Joint Laboratory for RNA Medicine, Sun Yat-Sen Memorial Hospital, Sun Yat-Sen University, Guangzhou 510120, China; Guangdong Provincial Key Laboratory of Malignant Tumor Epigenetics and Gene Regulation, Guangdong-Hong Kong Joint Laboratory for RNA Medicine, Sun Yat-Sen Memorial Hospital, Sun Yat-Sen University, Guangzhou 510120, China; Guangdong Provincial Key Laboratory of Malignant Tumor Epigenetics and Gene Regulation, Guangdong-Hong Kong Joint Laboratory for RNA Medicine, Sun Yat-Sen Memorial Hospital, Sun Yat-Sen University, Guangzhou 510120, China; Department of pharmacy, Sun-Yat-Sen Memorial Hospital, Sun Yat-sen University, Guangzhou 510120, China; Guangdong Provincial Key Laboratory of Malignant Tumor Epigenetics and Gene Regulation, Guangdong-Hong Kong Joint Laboratory for RNA Medicine, Sun Yat-Sen Memorial Hospital, Sun Yat-Sen University, Guangzhou 510120, China; Guangdong Provincial Key Laboratory of Malignant Tumor Epigenetics and Gene Regulation, Guangdong-Hong Kong Joint Laboratory for RNA Medicine, Sun Yat-Sen Memorial Hospital, Sun Yat-Sen University, Guangzhou 510120, China; Department of Urology, Sun Yat-Sen Memorial Hospital, Sun Yat-Sen University, Guangzhou 510120, China; Guangdong Provincial Key Laboratory of Malignant Tumor Epigenetics and Gene Regulation, Guangdong-Hong Kong Joint Laboratory for RNA Medicine, Sun Yat-Sen Memorial Hospital, Sun Yat-Sen University, Guangzhou 510120, China; Department of Urology, Sun Yat-Sen Memorial Hospital, Sun Yat-Sen University, Guangzhou 510120, China; Guangdong Provincial Key Laboratory of Malignant Tumor Epigenetics and Gene Regulation, Guangdong-Hong Kong Joint Laboratory for RNA Medicine, Sun Yat-Sen Memorial Hospital, Sun Yat-Sen University, Guangzhou 510120, China; Guangzhou Laboratory, Guangzhou International Bio Island, Guangzhou 510005, China; Cancer Research UK Nucleic Acid Structure Research Group, MSI/WTB Complex, The University of Dundee, Dow Street, Dundee DD1 5EH, UK; Guangzhou Laboratory, Guangzhou International Bio Island, Guangzhou 510005, China; Translational Research Institute of Brain and Brain-Like Intelligence and Department of Anesthesiology, Shanghai Fourth People's Hospital Affiliated to Tongji University School of Medicine, Shanghai 200434, China; Guangdong Provincial Key Laboratory of Malignant Tumor Epigenetics and Gene Regulation, Guangdong-Hong Kong Joint Laboratory for RNA Medicine, Sun Yat-Sen Memorial Hospital, Sun Yat-Sen University, Guangzhou 510120, China

## Abstract

Ribozymes are excellent systems in which to study ‘sequence - structure - function’ relationships in RNA molecules. Understanding these relationships may greatly help structural modeling and design of functional RNA structures and some functional structural modules could be repurposed in molecular design. At present, there is no comprehensive database summarising all the natural ribozyme families. We have therefore created Ribocentre, a database that collects together sequence, structure and mechanistic data on 21 ribozyme families. This includes available information on timelines, sequence families, secondary and tertiary structures, catalytic mechanisms, applications of the ribozymes together with key publications. The database is publicly available at https://www.ribocentre.org.

## Graphical Abstract

**Graphical Abstract:**
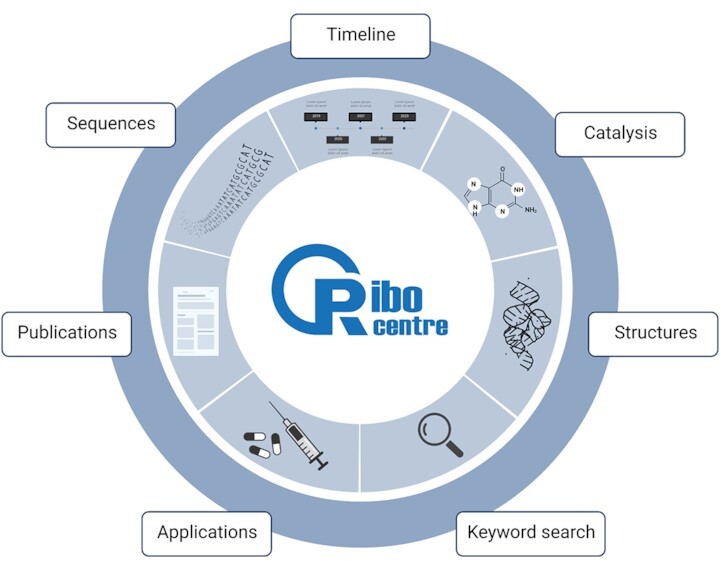
Ribocentre: a comprehensive database resource for ribozymes.

## INTRODUCTION

Ribozymes are functional RNA molecules that can catalyze chemical reactions, in addition to their roles as endonucleases (1), ribozymes play a variety of other roles critical to molecular biology (2), including the formation of peptide bonds in translation (3–5). They achieve impressive feats of catalysis, accelerating reactions by a million fold or greater (6). They are enzymes that comprise RNA, not protein. The first ribozymes were discovered in the 1980s in the labs of Cech and Altman (7–9), for which they were awarded the 1989 Nobel Prize in Chemistry for discovering ‘the catalytic properties of RNA molecules’.

The discovery of group I self-splicing introns and RNase P catalytic activity opened the era of RNA-based catalysis. During the ten years from 1980 to 1990, seven natural ribozyme families were identified, namely group I self-splicing introns (7), group II self-splicing introns (10), RNase P (11), hammerhead (12), hairpin (13), hepatitis delta virus (HDV) (14) and *Neurospora* Varkud satellite (VS) (15). During this period, the hypothesis of the spliceosome as a ribozyme was proposed based on the requirement of spliceosomal RNA and the similarity of the mechanism with group II self-splicing introns (16,17). From 1991 to 2013, only seven classes of ribozymes were found, specifically GIR1 (18), ribosomal RNA (19), glucosamine-6-phosphate riboswitch (glmS) (20), HDV-like ribozyme (21), long Interspersed Nuclear Element-1 (LINE-1) (21) and a natural ribozyme with 3' and 5' RNA ligase activity (22). In 2014 and 2015, using a new bioinformatic pipeline (23), the Breaker Lab identified the twister, twister-sister (TS), pistol and hatchet ribozymes (24). Hovlinc (human protein vlincRNA localization) is a recently evolved class of ribozymes, found in human ultra-long intergenic non-coding (vlinc) RNA (25). The discovery of ribozymes has changed the paradigm that proteins are the only catalytic molecules and lent considerable plausibility to the RNA world hypothesis in the origin of life on the planet (26). Some claims of ribozyme activity should be treated with caution, in particular for CoTC (27) and Vg1 (28). We are not aware of any publications confirming these activities, and it is highly probable that they are not ribozymes.

The range of chemical reactions catalysed by ribozymes is limited, but the existing ribozymes are widely distributed in nature. The hammerhead ribozyme was first discovered in the genomes of viruses and viroids where it is involved in the processing of RNA transcripts based on rolling circle replication (12). In addition, some hammerhead ribozymes exist in the introns of specific genes of amniotic animals and may participate in the processing of introns (29). The hairpin ribozyme originates from the negative strand of satellite RNA associated with the *tobacco ringspot* virus (sTRSV) (13). The twister ribozyme was identified in *Clostridium* and various *eukaryotes* (30).

The 3D structures of the majority of ribozymes have been determined, and the chemical mechanisms have been elucidated for some of these. A number of the known ribozymes carry out site-specific cleavage of RNA. It is possible that one can now design ribozymes to cleave virtually any RNA of interest, including viral RNAs, suggesting biomedical applications (31). In addition to roles as endonucleases, ribozymes play a variety of other functions critical to molecular biology, including the splicing of RNA transcripts, and the formation of peptide bonds in translation (7). The perhaps unexpected discovery that RNA could act as an enzyme opens the possibility that RNA might catalyse a much wider range of chemistry (currently studied by in vitro selection experiments as a kind of proof of principle) (32–35), and perhaps such RNA activities remain to be discovered in the contemporary biosphere.

RNA biology is extremely important, with multiple levels of riboregulation in cells. Moreover, the practical significance for human health has been highlighted by the developments in RNA viral inhibitors and mRNA vaccines during the pandemic. Ribozymes are good systems for understanding the ‘sequence - structure - function’ relationship of RNA molecules, since ribozymes are found in the genomes of species from all kingdoms of life and play a role in important reactions such as peptide-bond formation, RNA splicing, transfer RNA biosynthesis, and viral replication (8,10,12). This is therefore an excellent time to summarise these properties, and our new web-based database will make this generally accessible. Here, we provide the first ribozyme database, Ribocentre. This database can serve the scientific community as a resource, facilitating: (i) tabulation of the natural ribozymes and their properties; (ii) comparison of ribozyme sequence, structure and catalytic mechanism; (iii) the discovery of new ribozymes through comparative analysis.

## MATERIALS AND METHODS

### Database curation

The initial list of ribozymes to be displayed was summarised from reviews and publications in the past five years by PubMed (36) searching. At the same time, we conducted relevant searches on the Rfam database (37), Google search (https://www.google.com), RSCB PDB (38) and other websites using ribozyme as the keyword to proofread and supplement the list of ribozymes. The formation of the final ribozyme list was manually curated according to our research experience. PubMed and other search methods verify each other to ensure that the information is accurate.

Ribozyme sequences were downloaded from the Rfam database based on their Rfam IDs, followed by a further classification according to species.

The ribozyme structures were downloaded from the RCSB PDB for subsequent visualisation of structural aspects and generating the table of *Structure* page.

The information on *Ribozymes*, *Catalysis*, *Publications* and *Applications* pages was collected from PubMed. According to the characteristics of different aspects of ribozymes, the information is organized and used to make different ribozyme pages. The introduction content of ribozymes is cited from Wikipedia (https://www.wikipedia.org/) to provide some basic concepts of ribozymes. And the *News* section will update the information about recent publications, relative techniques, the latest updates of the database.

### Database implementation

Ribocentre has been implemented using Jekyll (https://jekyllrb.com/) which is a lightweight website generator based on the Ruby programming language coupled with Bootstrap (http://www.getbootstrap.com/) and jQuery (https://jquery.com/).

For the static structure illustration, the figures were generated by Adobe Illustrator, PyMOL (39) and KingDraw (http://www.kingdraw.cn/). For the interactive structure visualisation, fornac (40), which is an RNA secondary structure visualization tool using a force-directed graph layout, is used to display the secondary structures of the ribozymes directly in the website pages without installing software. Pdbe-molstar (41,42) plugin, a molecular 3D data viewer, is used to show an interactive 3D rendering of the ribozyme structures.

The web search function on the *Home* page is based on the google search engine (https://cse.google.com) for fast searching. The table search function is supported by several open-source Javascript libraries in the Datatables library (http://datatables.net), which make the data tables under the *Applications*, *Structures* and *Publications* pages allowing the data to be downloaded and printed. Google Sheets (https://www.google.com/sheets/about/) is used to collect feedback from website users. The *Help* page provides the process of how to feedback and some templates that can be used as a reference for users to provide feedback. Users can see all feedback records and our handling of issues, which is a better way to handle them than one-way submissions.

The website is mobile-friendly, allowing the pages to adjust to the user's screen size. In addition, the website is HTTPS-enabled, ensuring the security of data interaction between users and the website.

## DATABASE CONTENTS

The Ribocentre is a comprehensive natural ribozyme database of 16 886 sequences from 21 ribozyme families. With more than 290 descriptive reference articles, 165 experimentally determined structures and 48 different applications. The web pages are organised as follows:

The *Home* page of Ribocentre provides a brief introduction to ribozymes, a timeline of critical discoveries, example structures and enzymatic activities catalysed. Importantly, we provide a full site search function on the *Home* page. Users can search with keywords to access the ribozyme family of interest, while multiple example searches are supplied alongside the search box.

**Figure 1.**
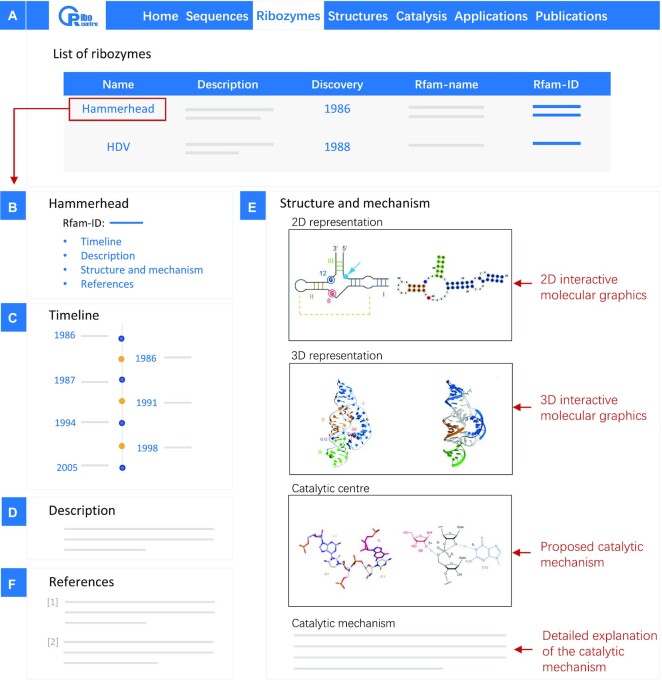
The *Ribozymes* page at Ribocentre. (**A**) A list of 21 ribozyme families are shown on the page. (**B**) Clicking on the ribozyme name brings up the information page for the chosen ribozyme. (**C–F**) The timeline, description, structure and mechanism, and references for the hammerhead ribozyme.

On the *Ribozymes* page, we list all the ribozyme families, showing the ribozyme name, a brief description, its year of discovery, and Rfam name and ID (Figure 1A). The name, year of discovery, and Rfam ID are shown in a bold blue with hyperlinks to further pages. For example, clicking on the ribozyme's name (*e.g*., hammerhead) will connect to the single entry page for that ribozyme (Figure 1B). The page for each ribozyme has four component panels: a timeline of results for that ribozyme (panel C), a description of the ribozyme (panel D), the structure and catalytic mechanism (panel E), and references (panel F). In the timeline, we have listed significant advances for each ribozyme, including initial discovery, sequence determination, identification of important structural domains of ribozymes, the first analysis of the 3D molecular structure, and elucidation of the catalytic mechanism. Users can click the year on the timeline to connect to PubMed to open the corresponding literature reference. Section E has sub-sections that present the secondary and tertiary structures, the structure of the catalytic centre and the current state of knowledge on the catalytic mechanism.

**Figure 2.**
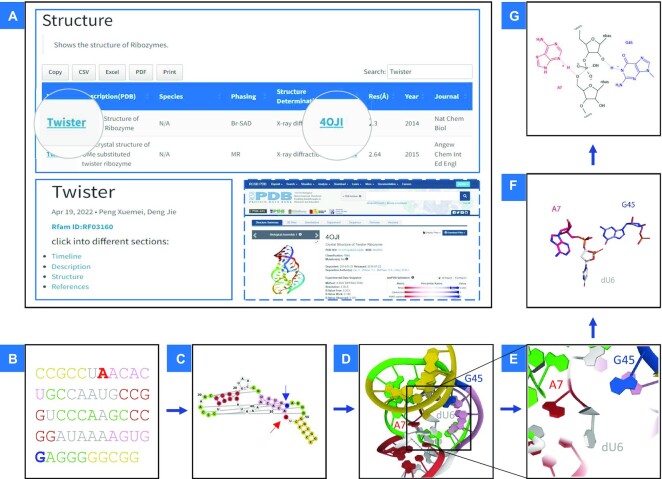
An example of the use of Ribocentre. Take the twister ribozyme as an example. (**A**) Enter ‘Twister’ on the *Structures* page to obtain all the twister ribozyme structure information. The PDB ID links to the PDB website (https://www.rcsb.org/). Click ‘Twister’ to connect to the twister ribozyme page. (**B**) The twister ribozyme comprises 54 nucleotides. (**C**) The general acid and base in that mediate the catalysis are shown in red and blue respectively on the secondary structure diagram. (**D**) The 2.3 Å twister ribozyme structure (PDB ID, 4OJI). (**E**) Zoom in to see the location of the catalytic acid and base. (**F**)The catalytic centre is shown in stick form. (**G**) The catalytic mechanism of the twister ribozyme is based upon nucleobase-mediated general acid-base catalysis.

The twister ribozyme provides an example of how to use the database as shown in Figure 2. Entering ‘Twister’ in the search box of the *Structures* page will display the parsed structure information, sorting the search results according to the resolution. Clicking the Protein Data Bank (PDB) ID connects to the relevant PDB page to provide more comprehensive information (Figure 2A). Similarly, clicking on the name of the ribozyme ‘Twister’ will link to the page for that ribozyme. On this page, users can obtain all the information relevant to the sequence, structure and catalytic mechanism of the twister ribozyme. To make it easier for users to understand the ribozyme from sequence to mechanism, we colour specific regions of a given ribozyme (e.g., twister) consistently at each stage. Users can use the Rfam ID to find the twister ribozyme sequence, which consists of 54 nucleotides (Figure 2B). Figure 2C shows the secondary structure displayed in the form of an interactive diagram. Long-range interactions are shown, and the ribozyme cleavage site is indicated by an arrow. Nucleobases acting as general acids and bases in the catalytic mechanism are highlighted in red and blue, respectively. Figure 2D shows the tertiary structure (PDB ID, 4OJI) at a resolution of 2.3 Å (43). The catalytic active centre is boxed, and the relative positions of the general acid and base can be seen. Users can zoom in on the structure to find the catalytic centre and the relative positions of the acid and base, as shown in Figure 2E, and the isolated components are shown in Figure 2F. Finally, a schematic depiction of the deduced chemical mechanism of catalysis is shown in Figure 2G (44). The Ribocentre provides users with an interactive pattern that makes it easier for them not only to understand the process by which nucleotides are folded to generate an active catalytic centre. In addition, this database provides information for researchers seeking to exploit ribozymes for laboratory and therapeutic applications.

The potential application of ribozymes in clinical research (45), metabolic engineering (46), plant breeding (47) and other fields has been recognized. As small RNA molecules with catalytic activity, ribozymes can be used as a safe and effective tool in molecular biology, which has attracted attention because of their potential specificity and lack of expected side effects. For example, shortly early in the study of ribozymes, the hammerhead ribozyme was applied as HIV gene therapy (48), the HDV ribozyme was used to produce homogenous RNAs with discrete 3' termini (49) and the twister ribozyme has been recently exploited as a highly versatile expression platform for artificial riboswitches (50). The database provides an *Applications* page that summarizes research on the application of ribozymes. Each application includes a brief description, reference and PubMed ID. The search box and download button allow the users to search and download the content of interest. We also provide examples in graphical form to show the real-world application scenarios for some particular ribozymes.

The different catalytic mechanisms of small ribozyme families and large ribozyme families are summarised on the *Catalysis* page. Most natural ribozymes carry out phosphoryl transfer reactions. The one exception to this is the peptidyl transfer reaction of the ribosome, where a nitrogen nucleophile attacks an sp^2^ hybridized carbonyl carbon atom to form a new C–N bond. In terms of the chemical mechanism of catalysis, the remaining ribozymes fall into two broad classes. The large ribozymes such as the self-splicing introns and RNase P are essentially metalloenzymes, using divalent metal ions to activate nucleophiles and organize and stabilize transition states. Thus, they use a similar mechanism to that of many nucleases such as restriction enzymes. By contrast, the small nucleolytic ribozymes use general acid-base catalysis to catalyse the phosphoryl transfer reaction initiated by the nucleophilic attack of the 2′-hydroxyl group. While the most common general base and acid used are nucleobases, nucleolytic ribozymes can use any of their functional groups, including hydrated metal ions and 2′-hydroxyl groups. Indeed, as a group, the nucleolytic ribozymes can be classified by the functionalities employed in general acid-base catalysis (51). Chemical mechanisms of some ribozymes such as the hairpin, VS and twister ribozymes are very well characterised, while some (such as TS) are more poorly understood at this time. We hope to update these as new experimental data become available. On the *Catalysis* page, we have arranged all the small ribozyme catalytic centers together in a diagram for users to compare intuitively, where red corresponds to acids and blue corresponds to bases. The mechanisms of large ribozymes are different, for example, the ribosome catalyzes peptide bond transfer, while RNase P catalyzes hydrolysis. All mechanisms of large ribozymes are also summarized in a chart.

Crystal structures have been solved for the majority of ribozymes except for some recent ribozymes such as LINE-1 and hovlinc. We summarize all existing structural information on the *Structures* page. Users can sort these data according to ribozyme name, species, PDB ID, resolution, structural analysis, method, year, and journal. Users can also view detailed information from the source of structural information by clicking the PDB ID, and clicking the ribozyme name to connect to the page of a given ribozyme. In addition, we also provide a search box and a download button on this page, so users can download the corresponding structural information for a chosen ribozyme.

Bibliographic information for each ribozyme can be found on the *Publications* page. To view specific literature, the user can click the journal link or click the name of the ribozyme to connect to the page of that ribozyme. The table can be sorted (ascending or descending) for the contents of any column by clicking on the column header. Users can search for the items by using the Search button option and typing the string of interest.

The database will be updated with the new progress of ribozyme research. Users are welcome to submit new ribozyme cases or related comments through the submission portal on the *Help* page to help us improve the database.

A feature of Ribocentre tables could be useful for users to find keywords or sequences by the search box on the right above the table. At the same time, the users could click buttons such as ‘CSV’, ‘EXCEL’ or ‘PDF’ and so on to save the information from the Ribocentre database. On *Catalysis*, *Applications* page, the database provides the download button for users to save the posters as a PDF file.

## DISCUSSION

The Ribocentre database provides users with an easy and flexible way to browse from lists of ribozymes and related publications over the past 40 years.

The *Ribozymes* pages include the research timeline, structural classification, molecular viewers, catalytic mechanism and other useful features. Through sequence searching the users may identify members of the wider ribozyme family and view the secondary and tertiary structures interactively. This emphasizes the correspondence between sequence and structure. The emphasis on ‘sequence - structure - function’ should give the user a greater understanding of its function and facilitate new ribozyme applications.

Ribocentre encourages users to use the available internal functions such as ordering, searching and interactive structure viewers and to send feedback and updated information, helping to ensure that data published on Ribocentre are updated, errors are corrected, and up-to-date links to external databases are maintained. Our database will be improved and remain fully functional and user-friendly.

## DATA AVAILABILITY

The web interface to the database is available at https://www.ribocentre.org. This website is free, open to all users and no login or password is required.
